# Solubility and thermodynamic study of deferiprone in propylene glycol and ethanol mixture

**DOI:** 10.1186/s13065-023-00950-1

**Published:** 2023-04-15

**Authors:** Samira Radmand, Homa Rezaei, Hongkun Zhao, Elaheh Rahimpour, Abolghasem Jouyban

**Affiliations:** 1 Student Research Committee, Faculty of Pharmacy, Tabriz University of Medical Sciences, Tabriz, Iran; 2 Pharmaceutical Analysis Research Center and Faculty of Pharmacy, Tabriz University of Medical Sciences, Tabriz, Iran; 3 College of Chemistry & Chemical Engineering, YangZhou University, YangZhou, 225002 Jiangsu People’s Republic of China; 4 Infectious and Tropical Diseases Research Center, Tabriz University of Medical Sciences, Tabriz, Iran; 5 Faculty of Pharmacy, Near East University, Nicosia, North Cyprus, PO BOX: 99138, Mersin 10, Turkey

**Keywords:** Deferiprone, Solubility, Binary solvent mixture, Cosolvency models, Thermodynamic properties

## Abstract

This work aims to obtain the solubility, density and thermodynamic parameters of deferiprone in propylene glycol and ethanol. For this purpose, a shake-flask technique was applied for solid–liquid equilibration and the spectrophotometry method was employed for solubility measurement. Solubility and density of deferiprone in non-aqueous mixtures of propylene glycol and ethanol were measured in the temperatures 293.2–313.2 K. Some equations including van’t Hoff, the Jouyban-Acree, the Jouyban-Acree-van’t Hoff, the mixture response surface and modified Wilson equations were used for the mathematical data modeling. The apparent thermodynamic parameters of the deferiprone dissolution process were computed and reported.

## Introduction

**Fig. 1:**
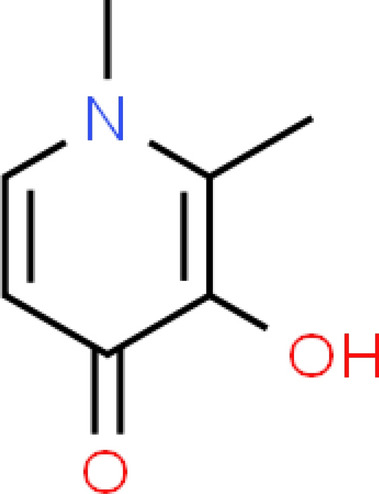
Molecular structure of deferiprone

Deferiprone (1,2-dimethyl-3-hydroxypyrid-4-one, Fig. 1) from alpha-ketohydroxpyridines family and as an iron chelator is mainly prescribed for thalassemia patients. Deferiprone has a high affinity toward iron with the capability to its eliminate from various parts of the body [1]. It is absorbed readily and stable in digestive system conditions. Moreover, deferiprone is also used for the treatment of leukemia, cancer, hemodialysis, and other diseases [2]. Solubility as an important physico-chemical property arises for each pharmaceutical compound and its knowledge is highly demanded in the selection of the best solvent or even antisolvent system [3]. Solubility data can be employed in various steps of the discovery and development of pharmaceutical compounds including synthesis, extraction, purification, sample preparation, analysis, etc. So, the solubility profile investigation in various mono/mixed solvents can assist to pharmacists, engineers, and chemists to choose a solvent or anti-solvent for desired application [4, 5]. Until now, the solubility of deferiprone has been studied in ethyl acetate, chloroform, acetonitrile, 1,4-dioxane and dichloromethane [6], ethanol, acetic acid, and sulfone [7], aqueous mixtures of ethylene glycol, propylene glycol (PG) and polyethylene glycol 400 [8], ethanol and N-methyl-2-pyrrolidone [9], and non-aqueous mixed solutions of ethanol + N-methyl-2-pyrrolidone [10]. However, deferiprone solubility has previously not been studied in PG and ethanol and the selected solvent and cosolvent for the current works are the most popular and routinely employed solvents in pharmaceutical companies.

With the aim of expanding of solubility database for deferiprone in solvent mixtures, the outcomes of work were (1) reporting the solubility and density for deferiprone in PG and ethanol with temperature increasing; (2) mathematical representation of data with some models; and (3) reporting the apparent thermodynamic properties of deferiprone dissolution.

## Experimental section

### Materials

Deferiprone (0.997 purchased from Arastoo Pharmaceutical Company, Tehran, Iran), PG (0.995, Merck, Darmstadt, Germany), and ethanol (0.999, Merck, Darmstadt, Germany) were the provided materials for mixed solvent preparation. Ethanol with a mass fraction purity of 0.935 (Jahan Alcohol Teb, Arak, Iran) and distilled water was employed for the dilution procedure.

### Solubility data

A shake-flask technique was applied for solid phase equilibration [11] and spectrophotometry was employed for solubility measurements. For the preparation of saturation solutions, excess amounts of drug were dispersed into a glass with 5 g of mono-solvents or solvent mixtures. After that, it was sealed and entered in an incubator (Kimia Idea Pardaz Azerbaijan (KIPA.co), Tabriz, Iran) with temperature control ability in the range of ± 0.2 K at ambient pressure on a shaker (Behdad, Tehran, Iran) for 48 h. After equilibration, the supernatant of solutions was centrifuged, diluted with ethanol: water (30:70% v/v), and their absorbance was measured with a spectrophotometer (Cecil BioAquarius CE 7250, UK) at 273 nm. The density for mixtures were also recorded using a 1.5 mL pycnometer with an uncertainty of 0.001 g∙cm^−3^.

### X-ray powder diffraction (XRD) analysis

The crystallinity of deferiprone (raw and residual in PG and ethanol) was studied by XRD analysis done on PHILIPS PW1730. The XRD data were provided from 10° to 80° (2θ) at 30 mA and 40 kV at atmospheric pressure.

### Mathematical models

The solubility measured in the current work were correlated with some linear cosolvency equations like the van’t Hoff [12] as a dependent model to temperature, the Jouyban-Acree and the Jouyban-Acree-van’t Hoff [13] as two models dependent to mass fraction of solvents and temperature, mixture response surface (MRS) [14] and non-linear model of the modified Wilson [15] as two models dependent to mass fractions of solvents which their equations were summarized here and the details for all of them were reported in our previous works.

1 $$lnx=A+\frac{B}{T}$$

2 $$ln{x}_{m}={\beta }_{1}{w}_{1}^{^{\prime}}+{\beta }_{2}{w}_{2}^{^{\prime}}+{\beta }_{3}\left(\frac{1}{{w}_{1}^{^{\prime}}}\right)+{\beta }_{4}\left(\frac{1}{{w}_{2}^{^{\prime}}}\right)+{\beta }_{5}{w}_{1}^{^{\prime}}. {w}_{2}^{^{\prime}}$$

3 $$ln{x}_{m,T}={w}_{1}ln{x}_{1,T}+{w}_{2}ln{x}_{2,T}+\frac{{w}_{1}.{w}_{2}}{T}\sum_{i=0}^{2}{J}_{i}.{({w}_{1}-{w}_{2})}^{i}$$

4 $$ln{x}_{m,T}={w}_{1}({A}_{1}+\frac{{B}_{1}}{T})+{w}_{2}({A}_{2}+\frac{{B}_{2}}{T})+\frac{{w}_{1}.{w}_{2}}{T}\sum_{i=0}^{2}{J}_{i}.{({w}_{1}-{w}_{2})}^{i}$$

5 $$-ln{x}_{m}=1-\frac{{w}_{1}[1+ln{x}_{1}]}{{w}_{1}+{w}_{2}{\lambda}_{12}}-\frac{{w}_{2}[1+ln{x}_{2}]}{{w}_{1}{\lambda}_{21}+{w}_{2}}$$

*x*_*m*_*, x*_*1*_ and *x*_*2*_ are solubilities in the mixed solvents, and mono solvents 1 and 2 and $w_{1}$ and $w_{2}$ are the mass ratios of solvents 1 (PG in this work) and 2 (ethanol in this work) in the absence of solute, respectively. *T* is the absolute temperature (K).

For studying the accuracy of the model, the mean relative deviation (*MRD* %) of the back-calculated data is obtained using the following equation.

6 $$MRD\%= \frac{100}{N}\sum \left(\frac{\left|Calculated\,Value-Observed\,Value\right|}{Observed\,Value}\right)$$

*N* is the number of data points. The statistical analysis was done by SPSS software version 16.0 [16] and all graphs were prepared using Microsoft Office Excel 2019 software.

### Hansen solubility parameters

Hansen solubility parameters were applied to study the solubilization power of the investigated system for deferiprone. The solubility parameter (*δ*) was reported by Hildebrand and Scott, and they noted that components with similar *δ* values are miscible [17]. As shown in Eq. (7), the second root of the solubility parameter is equal with the dividing the vaporization energy ($\Delta E$) by the molar volume ($V_{m}$):

7 $$\delta^{2} = \frac{{E_{coh} }}{{V_{m} }}$$

So, the solubility of two chemicals will be high, if their solubility parameters are close to each other. The solubility parameter of a component, based on Charles Hansen, ascribed to three parameters: dispersion forces ($\delta_{d}^{2}$), hydrogen bonds ($\delta_{h}^{2}$)and polar interactions ($\delta_{p}^{2}$) [18, 19].

8 $$\delta_{t}^{2} = \delta_{d}^{2} + \delta_{p}^{2} + \delta_{h}^{2}$$

The sum of three Hansen solubility parameters is resulted in Hildebrand parameter. Based on the solubility parameters, the dissolution tendency can be estimated. Using Eq. (9), difference of the Hansen solubility parameters of a cosolvent and a chemical solute is determined.

9 $$\Delta \delta_{i,j} = \sqrt {4\left( {\delta_{d}^{i} - \delta_{d}^{j} } \right)^{2} + \left( {\delta_{p}^{i} - \delta_{p}^{j} } \right)^{2} + \left( {\delta_{h}^{i} - \delta_{h}^{j} } \right)^{2} }$$

$\Delta \delta_{i,j}$ demonstrates the difference level and the *i* and *j* ascribed to the solvents and deferiprone, respectively [19]. Hoftyzer and Van Krevelen [20] introduced a technique for computing the partial solubility parameters of the organic compounds using group contributions. The equations for the computing of $\delta_{d}$,$\delta_{p}$ and $\delta_{h}$ are:

10 $$\delta_{d} = \frac{{\sum {F_{d} } }}{{V_{m} }}$$

11 $$\delta_{p} = \frac{{\sqrt {\sum {F_{p}^{2} } } }}{{V_{m} }}$$

12 $$\delta_{h} = \sqrt {\frac{{\sum {E_{h} } }}{{V_{m} }}}$$

where *F*_d_ and *F*_p_ correspond to the group contributions to the dispersion and the polar component, respectively, and *E*_h_ is hydrogen-bonding energy per structural group. The numerical values of *F*_*d*_, *F*_*p*_, and *E*_*h*_ of deferiprone are tabulated in Table 1 [20].

**Table 1:** The numerical values of solubility parameter component group contributions utilized in Hoftyzer and Van Krevelen’s method [20]

Structural Group	F_d_ (MJ/m^3^)^1/2^.mol^−1^	F_p_ (MJ/m^3^)^1/2^.mol^−1^	E_h_ J/mol
	20	800	5000
OH	210	500	20000
CH_3_	420	0	0
	70	0	0
– CO–	290	770	2000

The Hansen solubility parameters values for different mixtures used here in the absence of deferiprone (δ_mix_) was obtained using Eq. (13).

13 $$\delta_{mix} = \alpha \delta_{1} + (1 - \alpha )\delta_{2}$$

here α is volume fraction of PG in PG and water, δ_1_ and δ_2_ related respectively to the Hansen solubility parameters of neat PG and ethanol.

### Thermodynamic parameters

The Gibbs and van’t Hoff equations are employed for the investigation of the thermodynamics of deferiprone solubility in PG and ethanol mixture. The modified van’t Hoff model is:

14 $$\frac{\partial lnx}{\partial {\left(\frac{1}{T}-\frac{1}{{T}_{m}}\right)}_{p}}=-\frac{{\Delta H}^{^\circ }}{R}$$

*R* is the ideal gas constant [21] and* T*_*hm*_ is considered as the mean harmonic temperature computed as ${T}_{hm}=n/\sum_{i=1}^{n}(\frac{1}{T})$ (*n* is the number of studied temperatures). The slope and the intercept of ln *x vs* 1/*T* − 1/*T*_*hm*_ are employed to calculate $\Delta H^{ \circ }$ and $\Delta G^{ \circ }$, and $\Delta S^{ \circ }$values are calculated by Gibbs equation.

To assay the relative contributions of enthalpy (*ζ*_*H*_) and entropy (*ζ*_*TS*_) to $\Delta G^{ \circ }$ of deferiprone dissolution in the investigated mixtures, Eqs. (8) and (9) are used [22].

15 $${\zeta}_{H}=\frac{\left|{\Delta H}^{^\circ }\right|}{(\left|{\Delta H}^{^\circ }\right|+\left|{T\Delta S}^{^\circ }\right|)}$$

16 $${\zeta}_{TS}=\frac{\left|{T\Delta S}^{^\circ }\right|}{(\left|{\Delta H}^{^\circ }\right|+\left|{T\Delta S}^{^\circ }\right|)}$$

Furthermore, the following equations were applied to estimate the $\Delta_{mix} H^{ \circ }$ and $\Delta_{mix} S^{ \circ }$ mixing [23, 24].

17 $$\Delta_{sol} H^{ \circ } = \Delta_{fus} H^{303} + \Delta_{mix} H^{ \circ }$$

18 $$\Delta_{sol} S^{ \circ } = \Delta_{fus} S^{303} + \Delta_{mix} S^{ \circ }$$

where $\Delta_{fus} H^{303}$ and $\Delta_{fus} S^{303}$ are the thermodynamic parameters of fusion process at *T*_hm_ and obtained from Eqs. (19)–(21).

19 $$\Delta_{fus} H^{303} = \Delta_{fus} H^{{T_{fus} }} - \Delta C_{p} \left( {T_{fus} - T_{hm} } \right)$$

20 $$\Delta_{fus} S^{303} = \Delta_{fus} S^{{T_{fus} }} - \Delta C_{p} \ln \left( {\frac{{T_{fus} }}{{T_{hm} }}} \right)$$

21 $$\Delta C_{p} = \frac{{\Delta_{fus} H^{{T_{fus} }} }}{{T_{fus} }}$$

The values of $\Delta_{fus} H^{{T_{fus} }}$ and $T_{fus}$ for deferiprone were 32102.36 kJ mol^−1^ [25] and 545.15 K [26], respectively. The values were employed to compute the enthalpy and entropy change of fusion at *T*_hm_, *i.e*. $\Delta_{fus} H^{303}$ and $\Delta_{fus} S^{303}$ using Eqs. (12) and (13) and the values were 17.84 kJ mol^−1^ and 24.30 J mol^−1^ K^−1^, respectively.

The enthalpic ($\zeta_{H}^{mix}$) and entropic ($\zeta_{TS}^{mix}$) contributions to $\Delta_{mix} G^{ \circ }$ can be determined as:

22 $$\zeta_{H}^{mix} = \frac{{\left| {\Delta_{mix} H^{ \circ } } \right|}}{{\left| {\Delta_{mix} H^{ \circ } } \right| + \left| {T_{hm} \Delta_{mix} S^{ \circ } } \right|}}$$

23 $$\zeta_{TS}^{mix} = \frac{{\left| {T_{hm} \Delta_{mix} S^{ \circ } } \right|}}{{\left| {\Delta_{mix} H^{ \circ } } \right| + \left| {T_{hm} \Delta_{mix} S^{ \circ } } \right|}}$$

## Results and discussions

### XRD analysis

**Fig. 2:**
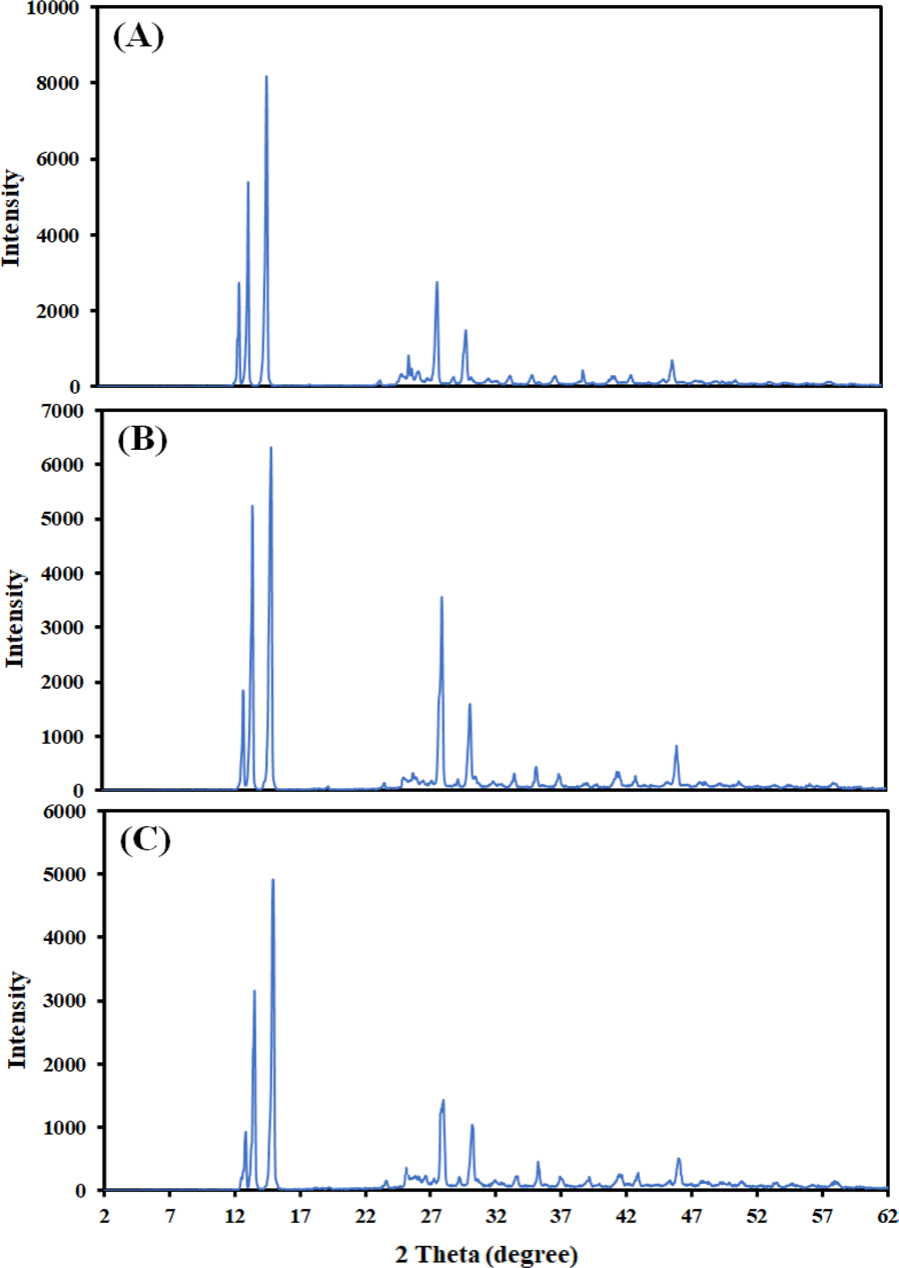
XRD pattern of raw deferiprone (**A**) and equilibrated deferiprone in ethanol (**B**) and PG (**C**)

Employing XRD equipment at room temperature and pressure, the XRD data of deferiprone residuals in mono-solvents were recorded and their patterns were given in Fig. 2. This analysis shows whether solid deferiprone in the saturated solutions form solvated compounds or polymorphs. As shown, the new characteristic peaks did not appear, showing that the crystallinity of deferiprone didnot change, and did not show polymorphic transformation in the dissolution process.

### Equilibrium solubility of deferiprone

**Fig. 3:**
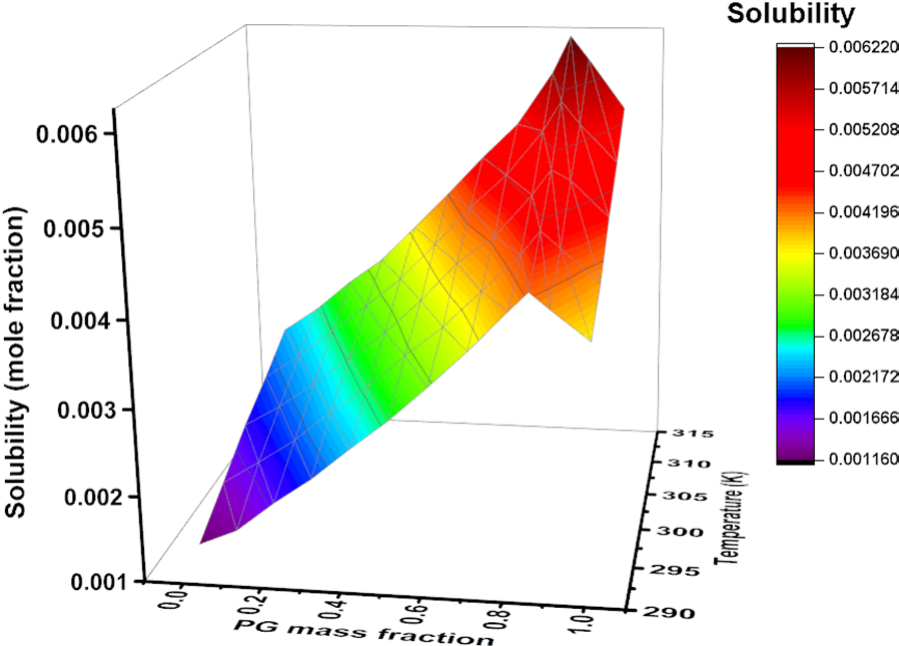
Solubility of deferiprone as a function of the mass fraction of PG and temperature

Solubility data of deferiprone in PG + ethanol were measured by a shake-flask technique. Table 2 tabulates the equilibrium mole solubility of deferiprone experimentally determined in the selected mixture within mass fraction composition (*w*_1_) ranging from 0.1 to 0.9. Moreover, the 3D plots for the solubility data were illustrated in Fig. 3. According to Table 2 and Fig. 3, the solubility profile of deferiprone was a function of the mass fraction of cosolvent and temperature. Deferiprone solubility monotonously increases with the temperature rising in all mixtures and increases with PG composition until 0.85, and then was followed by a decrease. These results show that the mixture with a PG mass fraction of 0.85 and ethanol mass fraction of 0.15 provides good conditions for deferiprone solubilization. This condition can be a combination of multiple factors such as polarity, van der Waals forces, preferential solvation, molecular shape and size, and other features of solute and solvent. The measured solubility data in neat PG (5.46 × 10^–2^) and ethanol (2.53 × 10^–2^) were close to those reported in the literature for PG (6.33 × 10^–2^) [8] and ethanol (1.84 × 10^–2^) [9] and a deviation between data were related to the person to person and procedure error.

**Table 2:** Experimental mole fraction solubility ($x_{m,T}^{{}}$) values as the mean of three measurements (± standard deviation) measured for deferiprone in the binary mixtures of PG and ethanol at different temperatures

*w*_1_^a^	293.2 K	298.2 K	303.2 K	308.2 K	313.2 K
0.00	1.17 (± 0.04) × 10^–3^	1.48 (± 0.01) × 10^–3^	1.80 (± 0.06) × 10^–3^	2.05 (± 0.06) × 10^–3^	2.33 (± 0.27) × 10^–3^
0.10	1.36 (± 0.03) × 10^–3^	1.76 (± 0.02) × 10^–3^	2.06 (± 0.10) × 10^–3^	2.35 (± 0.18) × 10^–3^	2.63 (± 0.04) × 10^–3^
0.20	1.71 (± 0.06) × 10^–3^	2.05 (± 0.02) × 10^–3^	2.37 (± 0.09) × 10^–3^	2.68 (± 0.17) × 10^–3^	3.00 (± 0.19) × 10^–3^
0.30	2.02 (± 0.07) × 10^–3^	2.42 (± 0.04) × 10^–3^	2.71 (± 0.05) × 10^–3^	3.03 (± 0.07) × 10^–3^	3.32 (± 0.09) × 10^–3^
0.40	2.39 (± 0.14) × 10^–3^	2.73 (± 0.07) × 10^–3^	3.04 (± 0.04) × 10^–3^	3.39 (± 0.23) × 10^–3^	3.76 (± 0.28) × 10^–3^
0.50	2.74 (± 0.02) × 10^–3^	3.13 (± 0.04) × 10^–3^	3.45 (± 0.04) × 10^–3^	3.84 (± 0.09) × 10^–3^	4.21 (± 0.35) × 10^–3^
0.60	3.14 (± 0.06) × 10^–3^	3.48 (± 0.10) × 10^–3^	3.92 (± 0.05) × 10^–3^	4.31 (± 0.43) × 10^–3^	4.68 (± 0.33) × 10^–3^
0.70	3.56 (± 0.02) × 10^–3^	3.91 (± 0.13) × 10^–3^	4.25 (± 0.10) × 10^–3^	4.72 (± 0.03) × 10^–3^	5.11 (± 0.50) × 10^–3^
0.80	4.02 (± 0.20) × 10^–3^	4.34 (± 0.03) × 10^–3^	4.75 (± 0.16) × 10^–3^	5.26 (± 0.16) × 10^–3^	5.76 (± 0.03) × 10^–3^
0.85	4.24 (± 0.33) × 10^–3^	4.62 (± 0.12) × 10^–3^	5.05 (± 0.25) × 10^–3^	5.62 (± 0.28) × 10^–3^	6.21 (± 0.09) × 10^–3^
0.90	4.06 (± 0.22) × 10^–3^	4.43 (± 0.10) × 10^–3^	4.93 (± 0.26) × 10^–3^	5.35 (± 0.18) × 10^–3^	5.93 (± 0.42) × 10^–3^
1.00	3.72 (± 0.42) × 10^–3^	4.04 (± 0.15) × 10^–3^	4.51 (± 0.17) × 10^–3^	4.90 (± 0.38) × 10^–3^	5.33 (± 0.47) × 10^–3^

^a^*w*_1_ is mass fraction of PG in the PG and ethanol mixtures in the absence of deferiprone

### Evaluation of mathematical models

Five well-known models (the van’t Hoff, the Jouyban-Acree, the Jouyban-Acree-van’t Hoff, the MRS, and the modified Wilson models) have been employed to carry out solubility modeling of deferiprone in the studied binary system and the model parameters were in Tables 3, 4, 5, 6, respectively. *MRD*% values were also shown in these Tables. Values of *MRD*% for the studied models were ranked as the van’t Hoff < the MRS < the modified Wilson < the Jouyban-Acree < the Jouyban-Acree-van’t Hoff that low *MRD*% values for all Eqs. (2.5%) indicating these models can provide satisfactory correlation solubility data in binary-solvents. Among these models, Jouyban-Acree and Jouyban-Acree-van’t Hoff models with correlation capability for all data in one run due to dependency on both mass fraction and temperature, provide a valuable model for solubility prediction. To check the prediction power of the Jouyban-Acree-van’t Hoff model, the minimum data number points (*i.e.* data in neat ethanol and PG at 293.2 and 313.2 K and solubility values in mass fractions of 0.3, 0.5, and 0.7 at 298.2 K) were correlated with the equation and trained model was obtained based on these data. And the rest of the data were calculated by the trained equation. *MRDs*% for predicted data were 3.5, 3.5, 4.0, 4.5 and 6.2 for 293.2, 298.2, 303.2, 308.2, and 313.2 K, respectively (overall *MRD* % was 4.3%).

**Table 3:** The van’t Hoff model parameters and the corresponding and *MRD*% for back-calculated deferiprone solubility data in the binary mixtures of PG and ethanol

*w*_1_	*A*	*B*	*MRD*%
0.00	3.983	− 3136.518	2.6
0.10	3.551	− 2962.427	3.3
0.20	2.385	− 2561.230	1.4
0.30	1.472	− 2242.954	1.8
0.40	1.008	− 2063.405	0.4
0.50	0.777	− 1954.916	0.6
0.60	0.583	− 1860.297	0.6
0.70	0.067	− 1673.268	0.4
0.80	0.177	− 1672.595	0.8
0.85	0.529	− 1759.766	0.7
0.90	0.415	− 1737.558	0.6
1.00	0.118	− 1675.779	0.5
Overall			1.1

**Table 4:** The MRS model constants at investigated temperatures and the *MRD*% for back-calculated deferiprone solubility data in the binary mixtures of PG and ethanol

*T* (K)	*β*_*1*_	*β*_*2*_	*β*_*3*_	*β*_*4*_	*β*_*5*_	*MRD*%
293.2	− 5.317	− 6.810	0^a^	− 0.005	0.726	1.5
298.2	− 5.245	− 6.547	0^a^	− 0.005	0.557	1.0
303.2	− 5.131	− 6.352	0^a^	− 0.005	0.337	1.0
308.2	− 5.034	− 6.217	0^a^	− 0.005	0.3020	1.1
313.2	− 4.880	− 6.062	0^a^	− 0.007	0^a^	1.5
Overall *MRD*%						1.2

^a^Not statistically significant (*p*-value > 0.05)

**Table 5:** Parameters calculated for the Jouyban-Acree, and Jouyban-Acree-van’t Hoff models and the *MRD*% for back-calculated deferiprone solubility data in the binary mixtures of PG and ethanol

	Jouyban-Acree		Jouyban-Acree-van’t Hoff	
PG + ethanol	J_0_	254.477	A_1_	0.118
	J_1_	245.335	B_1_	− 1675.779
	J_2_	286.712	A_2_	3.983
			B_2_	− 3136.518
			J_0_	254.310
			J_1_	245.645
			J_2_	286.320
*MRD*%	2.2		2.5	

^a^Not statistically significant (*p*-value > 0.05)

**Table 6:** The modified Wilson model parameters at the investigated temperatures and the *MRD*% for back-calculated deferiprone in the binary mixtures of PG and ethanol

*T* (K)	λ_12_	λ_21_	*MRD*%
293.2	2.294	0.647	1.7
298.2	2.279	0.631	1.7
303.2	2.521	0.559	1.2
308.2	2.629	0.542	1.4
313.2	2.911	0.495	1.5
Overall			1.5

In the next part, the saturated solution’s density was determined and correlated with the Jouyban-Acree model. The trained equation was as:

24 $$ln{\rho }_{m,T}={w}_{1}ln{\rho }_{1,T}+{w}_{2}ln{\rho }_{2,T}-1.712\frac{{w}_{1}.{w}_{2}}{T}$$

$\rho_{m,T}^{{}}$ is the density of solute saturated mixtures and $\rho_{1,T}^{{}}$, and $\rho_{2,T}^{{}}$ are solute density saturated mono-solvent at temperature *T*. The back-calculated *MRD*% for these data is 0.1% showing that the Jouyban-Acree equation possesses a good power for prediction of density at various temperatures. The measured density (g.cm^–3^) of deferiprone-saturated mixtures at various temperatures were tabulated in Table 7.

**Table 7:** Measured density (g.cm^–3^) of deferiprone saturated solutions in the binary mixtures of PG and ethanol at different temperatures

*w*_1_	293.2 K	298.2 K	303.2 K	308.2 K	313.2 K
0.00	0.789 ± 0.001	0.788 ± 0.001	0.788 ± 0.002	0.784 ± 0.001	0.782 ± 0.001
0.10	0.811 ± 0.001	0.810 ± 0.001	0.809 ± 0.001	0.806 ± 0.001	0.804 ± 0.001
0.20	0.833 ± 0.001	0.832 ± 0.001	0.830 ± 0.001	0.828 ± 0.001	0.826 ± 0.001
0.30	0.855 ± 0.001	0.855 ± 0.001	0.853 ± 0.001	0.850 ± 0.001	0.849 ± 0.001
0.40	0.881 ± 0.005	0.877 ± 0.001	0.876 ± 0.001	0.873 ± 0.001	0.871 ± 0.001
0.50	0.905 ± 0.001	0.902 ± 0.001	0.901 ± 0.001	0.900 ± 0.001	0.900 ± 0.001
0.60	0.929 ± 0.001	0.924 ± 0.001	0.923 ± 0.001	0.922 ± 0.001	0.922 ± 0.001
0.70	0.955 ± 0.002	0.951 ± 0.001	0.950 ± 0.001	0.949 ± 0.001	0.945 ± 0.001
0.80	0.982 ± 0.002	0.978 ± 0.001	0.976 ± 0.001	0.975 ± 0.001	0.974 ± 0.002
0.85	0.995 ± 0.001	0.992 ± 0.002	0.992 ± 0.001	0.990 ± 0.001	0.988 ± 0.001
0.90	1.008 ± 0.001	1.006 ± 0.002	1.004 ± 0.001	1.003 ± 0.001	1.000 ± 0.001
1.00	1.036 ± 0.001	1.035 ± 0.001	1.034 ± 0.001	1.032 ± 0.001	1.029 ± 0.002

### Hansen solubility parameters results

The Hansen solubility parameters for deferiprone were computing by the given method by Hoftyzer and Van Krevelen [20] and for pure solvents of ethanol and PG were taken from Ref. [27]. The results were given in Table 8. Furthermore, δ_mix_ values for various PG and ethanol mixtures were found as 26.8 to 29.7 MPa^1/2^. As shown, the Hansen solubility parameters values of binary systems with 0.5 < *w*_1_ < 0.8 (δ_mix_ = 28.1 to 29.3 MPa^1/2^) have similar to that of deferiprone (δ = 27.9 MPa^1/2^) which in acceptable agreement with measure solubility data.

**Table 8:** Solubility parameter for the used materials along with the values of *Δδ* for deferiprone as a solute and each solvent

Materials	*δ*_*d*_ (MPa^1/2^)	*δ*_*p*_ (MPa^1/2^)	*δ*_*h*_ (MPa^1/2^)	*δ*_*t*_ (MPa^1/2^)
Ethanol	15.8	8.8	19.4	26.5
PG	16.8	9.4	23.3	30.2
Deferiprone	14.0	13.4	20.0	27.9
Hansen solubility parameters for different PG + ethanol mixtures				
*w*_1_			*δ*_*mix*_ (MPa^1/2^)	
0.0			26.5	
0.1			26.8	
0.2			27.1	
0.3			27.4	
0.4			27.7	
0.5			28.1	
0.6			28.5	
0.7			28.9	
0.8			29.3	
0.85			29.5	
0.9			29.7	
1.0			30.2	

### Thermodynamic calculations

$\Delta H^{ \circ }$, $\Delta S^{ \circ }$and $\Delta G^{ \circ }$, and for deferiprone dissolution procedure in investigated mixtures were calculated as explained in Sect. 2.5. ∆*H* values were positive and showed a maximum value (26.10 kJ.mol^−1^) at *w*_1_ = 0.0 and the minimum value (13.87 kJ.mol^−1^) at *w*_1_ = 0.7. ∆*S* values were also positive showing the entropy-driven mechanism of the dissolution procedure. ∆*G* values decreased from 13.30 to 16.04 kJ.mol^−1^ and show a minimum amount in solution with a high solubility value for deferiprone. *ζ*_*H*_ and *ζ*_*TS*_ were also shown in Table 9 as relative contributions of *ΔH* and *TΔS* to ∆*G.*

**Table 9:** Apparent thermodynamic parameters for dissolution behavior of deferiprone in the binary mixtures of PG and ethanol at *T*_*hm*_

*w*_1_	Δ*G*° (kJ.mol^–1^)	Δ*H*° (kJ.mol^–1^)	Δ*S*° (J.K^–1^.mol^–1^)	*T*Δ*S*° (kJ.mol^–1^)	*ζ*_*H*_	*ζ*_*TS*_
0.00	16.04	26.10	33.21	10.06	0.722	0.278
0.10	15.68	24.58	29.36	8.90	0.734	0.266
0.20	15.29	21.27	19.73	5.98	0.781	0.219
0.30	14.94	18.68	12.34	3.74	0.833	0.167
0.40	14.61	17.15	8.36	2.53	0.871	0.129
0.50	14.29	16.17	6.19	1.87	0.896	0.104
0.60	14.00	15.48	4.90	1.48	0.913	0.087
0.70	13.74	13.87	0.43	0.13	0.991	0.009
0.80	13.46	13.90	1.44	0.44	0.970	0.030
0.85	13.30	14.66	4.48	1.36	0.915	0.085
0.90	13.40	14.41	3.33	1.01	0.935	0.065
1.00	13.64	13.92	0.94	0.28	0.980	0.020

**Fig. 4:**
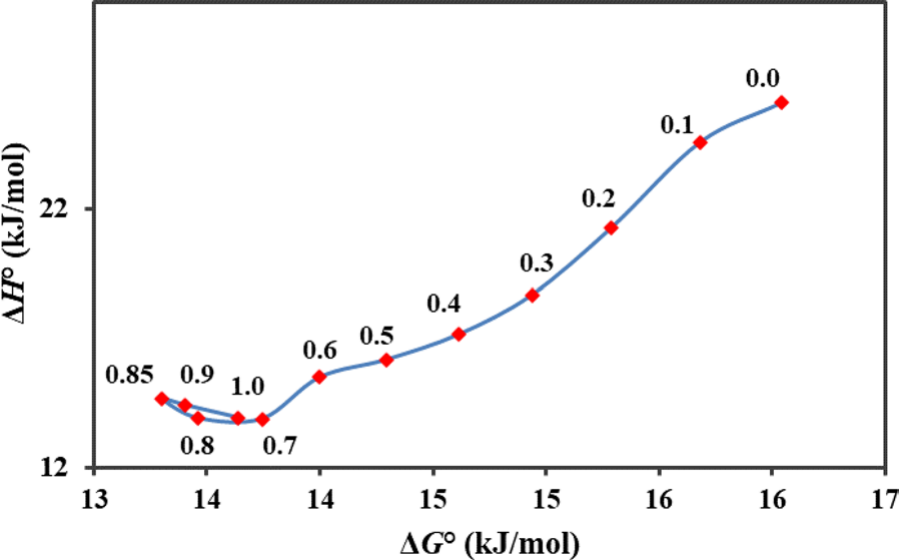
Enthalpy-entropy compensation plot for deferiprone in the non-aqueous mixtures of PG and ethanol at 303.0 K. The points represent the mass fraction of PG in PG and ethanol mixtures in the absence of solute

The plot of ∆*H* vs ∆*G* was used for finding the cosolvency mechanism for the investigated mixtures. As shown in Fig. 4, a region with a negative slope in 0.7 ≤ *w*_1_ ≤ 1.0 indexing entropy-driven mechanism and a region with a positive slope in 0.0 ≤ *w*_1_ ≤ 0.7 indexing an enthalpy-driven mechanism.

**Fig. 5:**
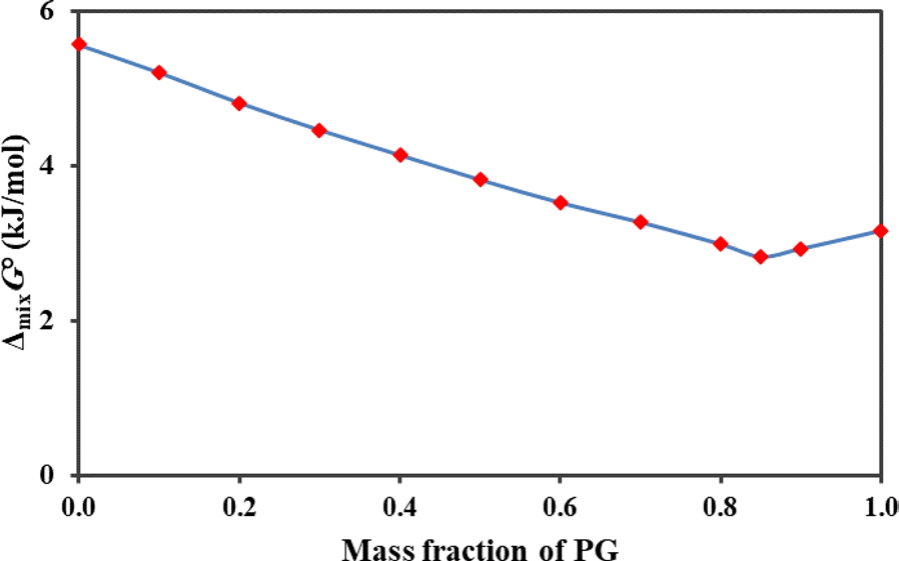
The Δ_mix_G^∘^ values relative to mixing process of deferiprone in PG/ethanol system at T_hm_ = 303.0 K

Moreover, the thermodynamic parameters of mixing for deferiprone solubility in the investigated system were given in Table 10. Using analysis of the partial contributions by ideal solution (related to solute fusion procedure) and mixing procedures to the enthalpy and entropy of the mixture, it was found that Δ_fus_H (303) and Δ_fus_S (303) were positive (17.84 kJ mol^−1^ and 24.30 J mol^−1^ K^−1^, respectively). Δ_mix_H^∘^ values were positive in ethanol-rich mixtures and were negative with increasing the PG mass fraction. The neat change of Δ_mix_H^∘^ values was in the results of the contribution of various interactions: (a) the enthalpy of cavity formation was endothermic owing to the required energy to overcome the cohesive forces of the solvent that reduces the drug solubility and (b) the enthalpy of solvent—solute interaction was exothermic and it was resulted of the van der Waals and Lewis acid–base interactions [28]. The placing of water molecules surrounding the nonpolar groups of solutes (hydrophobic hydration) ascribed to reduce the neat mixing heat to low or negative values in aqueous mixtures. The Δ_mix_S^∘^ values have negative values at higher mass fraction of PG. The pattern of Δ_mix_G^∘^ values were given in Fig. 5, according to that, the Δ_mix_G^∘^ values of system decrease with rising PG mass fraction reaching a negative value at *w*_*1*_ = 0.85 that show the highest values of deferiprone solubility.

**Table 10:** Thermodynamic functions relative to mixing process of deferiprone in the investigated mixtures at 303 K

*w*_1_	Δ_mix_*G*° (kJ.mol^–1^)	Δ_mix_*H*° (kJ.mol^–1^)	Δ_mix_*S*° (J.K^–1^.mol^–1^)	*T*Δ_mix_*S*° (kJ.mol^–1^)	*ζ*_*H*_	*ζ*_*TS*_
0.00	5.56	8.26	8.90	2.70	0.754	0.246
0.10	5.20	6.74	5.06	1.53	0.815	0.185
0.20	4.81	3.42	− 4.57	− 1.39	0.712	0.288
0.30	4.46	0.83	− 11.96	− 3.62	0.187	0.813
0.40	4.13	− 0.70	− 15.95	− 4.83	0.126	0.874
0.50	3.81	− 1.67	− 18.11	− 5.49	0.234	0.766
0.60	3.52	− 2.36	− 19.40	− 5.88	0.287	0.713
0.70	3.26	− 3.97	− 23.87	− 7.23	0.354	0.646
0.80	2.98	− 3.95	− 22.86	− 6.93	0.363	0.637
0.85	2.82	− 3.19	− 19.82	− 6.01	0.347	0.653
0.90	2.92	− 3.43	− 20.97	− 6.35	0.351	0.649
1.00	3.16	− 3.92	− 23.37	− 7.08	0.357	0.643

## Conclusions

Herein, solubility for deferiprone in PG and ethanol mixture at five temperatures was measured and correlated with some cosolvency equations. The *MRDs*% calculated for back-calculated data for these equations were in the range of 1.1–2.5%. Calculation of thermodynamic parameters showed that the deferiprone dissolution in the investigated mixtures was endothermic and facilitated in a higher concentration of PG (*w*_1_ = 0.85).

## Data Availability

The datasets used and/or analysed during the current study are available from the corresponding author on reasonable request.
